# Hepatitis B, C and human immunodeficiency virus knowledge among the general greek population: results from the Hprolipsis nationwide survey

**DOI:** 10.1186/s12889-022-14353-9

**Published:** 2022-11-05

**Authors:** Sylvia Kaskafetou, Argiro Karakosta, Vana Sypsa, Natasa Kalpourtzi, Magda Gavana, Apostolos Vantarakis, George Rachiotis, Grigoris Chlouverakis, Grigoris Trypsianis, Paraskevi V. Voulgari, Yannis Alamanos, George Papatheodoridis, Giota Touloumi, on behalf of the Hprolipsis study group, Agis Terzidis, Tzanetos Antypas, Christina Psara, Theofilos Rozenberg, Maria Kantzanou

**Affiliations:** 1 Dept of Hygiene, Epidemiology, & Medical Statistics, Medical School, National and Kapodistrian University of Athens, 75 Mikras Asias Street, 11527 Athens, Greece; 2 Dept of Primary Health Care, General Practice and Health Services Research, Medical School of Aristotle University, Thessaloniki, Greece; 3 Public Health, Medical School, University of Patras, Patra, Greece; 4 Dept of Hygiene and Epidemiology, Medical Faculty, University of Thessaly, Larisa, Greece; 5 Lab of Biostatistics, School of Medicine, University of Crete, Crete, Greece; 6 Lab of Medical Statistics, Medical School, Democritus University of Thrace, Thrace, Greece; 7 Dept of Internal Medicine, Medical School, University of Ioannina, Ioannina, Greece; 8 Inst of Epidemiology, Preventive Medicine and Public Health, Corfu, Greece; 9 Dept of Gastroenterology, Medical School, National and Kapodistrian University of Athens, Athens, Greece

**Keywords:** HCV, HBV, HIV, Knowledge, Risk factors, Health survey

## Abstract

**Background:**

Although several studies on hepatitis B (HBV), C (HCV) and human immunodeficiency virus (HIV) infection have been conducted in Greece, little is known on the knowledge level of the Greek population towards these three infections. Our aim was to assess the knowledge level of the adult Greek general population about the HBV, HCV and HIV.

**Methods:**

Data were derived from the first general population health survey, Hprolipsis. The sample was selected by multistage stratified random sampling. A standardized questionnaire was administered by trained interviewers during home visits. A knowledge score was constructed based on responses to 17 per infection selected items and categorized in three levels; high (12–17 correct replies) medium (6–11) and low (0–5). Among 8,341 eligible individuals, 6,006 were recruited (response rate: 72%) and 5,878 adults (≥ 18 years) were included in the analysis. The statistical analysis accounted for the study design.

**Results:**

Only 30.4%, 21.6%, and 29.6% of the participants had a high overall knowledge level of HBV, HCV and HIV, respectively. These low percentages were mainly attributed to the high levels of misconception about transmission modes (65.9%, 67.2%, and 67.9%, respectively). Results showed that increasing age and living out of the big metropolitan cities were associated with decreased odds of having higher knowledge. Female gender, higher education level, higher monthly family income, higher medical risk score, history of testing and being born in Greece or Cyprus, were associated with increased odds of having higher knowledge.

**Conclusions:**

There are significant knowledge gaps in the Greek general population regarding modes of transmission, preventive measures and treatment availability for HBV, HCV and HIV. There is an urgent need for large scale but also localized awareness activities targeted to less privileged populations, to fill the gaps in knowledge and increase population engagement in preventive measures.

**Supplementary Information:**

The online version contains supplementary material available at 10.1186/s12889-022-14353-9.

## Background

Despite substantial improvements in treating hepatitis B (HBV) and human immunodeficiency virus (HIV) and curing hepatitis C virus (HCV), these three infections remain of important public health concern generating excessive financial and societal costs [1–3]. It is estimated that in 2019, about 296 million people were living with chronic HBV and 58 million with chronic HCV infection worldwide, contributing jointly up to 1.1 million deaths in 2019, while about 38 million were living with HIV, contributing around 690,000 deaths in 2019 [4]. Despite global efforts to control these 3 infections, in 2019, 1.5 million people were newly infected with hepatitis B, 1.5 million with hepatitis C whereas 1.7 million people acquired HIV [4].

All three infections share similar routes of transmission whereas they are characterized by a long asymptomatic period with symptoms occurring at late stages, particularly among those infected with viral hepatitis B and C, leading thus to delayed diagnosis and treatment [5–7]. The absence of widespread screening programs also adds to late diagnosis. It is estimated that globally only 10% of those with chronic hepatitis B knew their status in 2019, of which 22% received treatment; the corresponding figures for chronic hepatitis C were 21% and 63% [4]. Following global efforts to achieve the 90–90-90 goal for HIV infection (90% of those living with HIV (PLHIV) be diagnosed, 90% of diagnosed been treated and 90% of those treated being virally suppressed) by 2020, the global situation in 2019 was: 81% of the PLHIV were diagnosed, 83% of those diagnosed were treated and 88% of those treated achieved viral suppression [4] whereas late presentation (i.e., CD_4_ cell counts at diagnosis < 350 cells/µl) remains as high as 50% [8, 9].

Access to prevention, diagnosis and treatment is limited by a lack in understanding and evidence-based knowledge as well as by social stigma and discrimination [10]. It has been shown that hepatitis and HIV knowledge is associated with disease management, outcomes and risk perception [11, 12]. Lack of knowledge and especially misconceptions regarding routes of transmission of these three infections leads to persisting social stigma and discrimination [13–15]. Increasing knowledge seems to lead to significant reductions in discriminatory attitudes and improvement in rates of disease screening and HBV vaccination and, consequently, to decrease in the incidence of these infections [15–19].

In Greece, although several studies on HBV, HCV and HIV infection have been conducted in specific population groups, data on general populations’ knowledge level and misconceptions as well as on behaviors associated with these infections are limited [20–25]. Such data though are of high public health importance, particularly in the light of the recent treatment advances; identifying knowledge gaps and associated factors are essential to design and implement targeted public health interventions. Taking advantage of the National health examination survey Hprolipsis (design and development of viral hepatitis and HIV infection screening program in the general population and vulnerable populations) we aimed to assess knowledge level and misconceptions on HBV, HCV and HIV infection, as well as behaviors associated with these infections in a representative sample of the general adult Greek population.

## Material and methods

### The Hprolipsis survey

Data were derived from the Hprolipsis health examination survey [26]. Hprolipsis is the first epidemiological survey on HBV, HCV, and HIV infections in three adult (≥ 18 years) populations in Greece (general population, migrants, and Greek Roma). Hprolipsis was nested within EMENO health examination survey [27]. In this paper, only data from the general population are analyzed. For the general population, a multistage random sample was drawn based on the 2011 Census. Data on the general population were collected during home visits, where trained interviewers administered a standardized questionnaire to study participants, whereas trained physicians performed physical examinations and collected blood samples using standardized procedures and equipment. Hprolipsis survey was approved by the Ethics Committee of the National and Kapodistrian University of Athens (date: March 4, 2015, protocol: 6141). Details on the study design can be found elsewhere [26].

### Measurements

#### Knowledge of HBV, HCV and HIV

For assessing the knowledge level of HBV, HCV and HIV, 17 “true/false” statements for each infection were administered. The first seven statements concerned general knowledge about the infections, whereas the next ten concerned possible transmission modes. Five of these ten statements were correct indicating the routes the infections could be transmitted whereas the other five were false, indicating misconceptions about transmission. “Don’t know” and “don’t answer” options were added to prevent participants from randomly guessing the answer, thereby inflating the percentage of correct answers. In Annex 1, the 17 statements and their correct answers are presented.

Three different knowledge scores were created: a) overall knowledge score that evaluates all 17 statements; b) transmission modes knowledge score that evaluates the 5 statements referring to the true routes of transmission (questions 8 to 12 in Annex 1) and c) misconceptions score that evaluates the 5 statements referring to false transmission routes (questions 13 to 17 in Annex 1). To construct the knowledge scores, a point was given to each correct answer; that is, the overall knowledge score ranged from 0–17, whereas the other two scores ranged from 0–5. For all three scores, answers “I don’t know” and “I don’t answer” were considered as wrong replies and they did not contribute to the scores.

Additionally, a total knowledge over all three infections scores (referred from now on as total overall knowledge score) was generated as the sum of the overall knowledge scores of each infection; thus, it ranged from 0 to 51.

### Risk factors score

In the international literature, a variety of risk factors associated with knowledge and behavior toward the three infectious diseases have been proposed [17, 18, 28–31]. In this survey, we created two scores of risk factors: the behavior score that refers to behaviors associated with these infections and the medical transmission risk score.

Behaviors associated with these infections are defined as behaviors correlated with increased risk of infection. For developing the behavior score, the following factors were considered:

1. Tattoo or body piercing
2. Injected drugs use
3. More than 5 new sexual partners during the last year
4. Not usually using condom
5. Other sexually transmitted disease diagnosis

The behavior score was created as the number of these behaviors coexisting in one person and was further categorized in three levels: low level (no such factors), moderate level (one factor), and high level (two or more factors co-existed). History of injecting drug use was categorized as high level independently of the co-existence of other factors.

For medical risk, medical practices associated with increased risk of infection were considered. Namely:

1. Occupational risk: contact with blood, blood products, syringe or needles at work
2. Family medical history: family member with HBV or HCV infection
3. Surgery with narcosis or endoscopy
4. Transfusion before 1992
5. Hemodialysis
6. Transplantation

A score was created as the number of risk factors coexisting in one person. Three levels of medical risk were considered: low medical risk (no risk factors), moderate medical risk (one risk factor), and high medical risk (two or more factors).

For both, medical and behavior risk score, “I don’t know/don’t answer” were treated as missing. The factors to be considered in behavior and medical scores were based on relevant literature review and expert’s opinion; cut-offs for grouping were based on scores’ distribution as well as on experts’ opinion.

### Statistical analysis

Participants who had missing age or missing in at least six questions out of the total 51 knowledge questions across all three infections (i.e., ≥ 10% of the questions missing), were excluded from the analysis. For participants who had missing values for up to 5 knowledge questions, their missing responses were replaced by the average infection-specific score, based on their replies to the rest of the infection-specific questions. Median (IQR) knowledge scores were calculated. In addition, all knowledge scores were classified in three levels (low, moderate, and high) by allowing each level to have equal number of correct answers. That is, the overall knowledge was classified as low if the score was 0–5, moderate if it was 6–11 and high if it was 12–17. The corresponding cut-off points were 0–1, 2–3 and 4–5 for the transmission mode and for the misconceptions score.

For the statistical analysis, the study sampling weights were taken into account, with additional weighting to match the age, gender and geographical distribution of the sample to that of the Greek population based on the 2011 census provided by the National Statistics Agency (post-stratification weighting). Weighted means and standard deviations or median and IQR for continuous variables and weighted percentages for categorical variables were provided.

To assess the factors associated with levels of knowledge, weighted multivariable ordinal logistic regression models were fitted using as dependent variable the ordinal (low, moderate, high) total overall knowledge score. Estimates’ standard errors (SEs) were obtained using the Taylor linearization method. Participants were classified as having low total overall knowledge score if their score was 0–16, moderate if their score was 17–31 and high if their score was 34–51. Several demographic, socioeconomic and clinical factors were evaluated for their association with knowledge levels. Missing values in these cofactors were imputed using multiple imputations (MI) by the chained equations method (MICE) [32] under the assumption that data were, conditionally on observed information, missing at random. Partially observed cofactors were alcohol consumption, smoking status, income, educational level, unemployment, ever tested for infectious diseases, having kids, birthplace, having a chronic disease and all the variables involved in the calculation of the medical risk score. Completely observed cofactors were gender, area of residence, age and degree of urbanization. Ten imputed datasets were created with a burn-in period of 300 iterations. For the main analysis, the imputed data were used. As a sensitivity analysis, complete case analysis was also carried out.

All statistical analyses were performed using the statistical software STATA (version 13.0).

## Results

### Descriptive characteristics

In total, 8341 individuals were invited to participate in the study, and 6,006 individuals were finally recruited (response rate: 72.0%). Thirteen individuals with unknown age and 115 (1.9%) with missing values at ≥ 6 (105 with missing at ≥ 25) out of the 51 knowledge questions, were excluded, leading to a total of 5,878 participants included in the analysis.

Descriptive characteristics of the participants after post stratification weighting are presented in Table 1. The mean age of the population was 47.7 years; 51.5% were women, 47.2% had graduated secondary or postsecondary education and 61.4% were married or in cohabitation. About 88.0% were born in Greece/Cyprus and 11.3% in a country other than Greece/Cyprus. Household monthly income was up to 900€ for 39.9% of the population, whereas 15.3% were unemployed, a result that reflects the fact that the survey was conducted during the financial crisis in Greece (2013 – 2016).

**Table 1:** Socio-demographic characteristics of study participants (*N* = 5,878). Weighted percentages and median (IQR) are presented

**Gender, n (%)**^a^	
Male	2494 (48.5)
Female	3384 (51.5)
**Age, years**^a^	
Median (IQR)	47,7 (34, 64)
**Educational level, n (%)**^a^	
Up to primary	2083 (28.9)
Up to secondary/post-secondary	2567 (47.2)
University or higher	1189 (23.3)
Other/ Unknown	39 (0.6)
**Family status, n (%)**^a^	
Married/ in cohabitation	3890 (61.4)
Single	1968 (38.2)
Unknown/no answer	20 (0.4)
**Household income/month, n (%)**^a^	
Up to 900€	2372 (39.9)
900€—1700€	1640 (28.3)
> 1700€	601 (11.0)
Unknown	1265 (20.8)
**Employment status, n (%)**^a^	
Employed/ non unemployment	5076 (84.2)
Unemployment	771 (15.3)
Unknown	31 (0.5)
**Country of Birth, n (%)**^a^	
Greece/Cyprus	5262 (88.0)
Other	573 (11.3)
Unknown	43 (0.7)

^a^weighted percentages or weighted median (IQR)

### Knowledge score

In total, 289 (4.9%) had missing in just one knowledge question, 61 (1%) in 2 questions and 57 (0.97%) to 3–5 questions out of the 51 total questions. The responses to individual knowledge questions are summarized in Annex 1. It is notable the high percentage of participants replying “I don’t know” in most knowledge questions as well as the high percentages of people believing that the three infections can be transmitted through social contact (drinking/eating from the same utensils; using the same toilet, by kissing and by mosquitos’ bite).

**Fig. 1:**
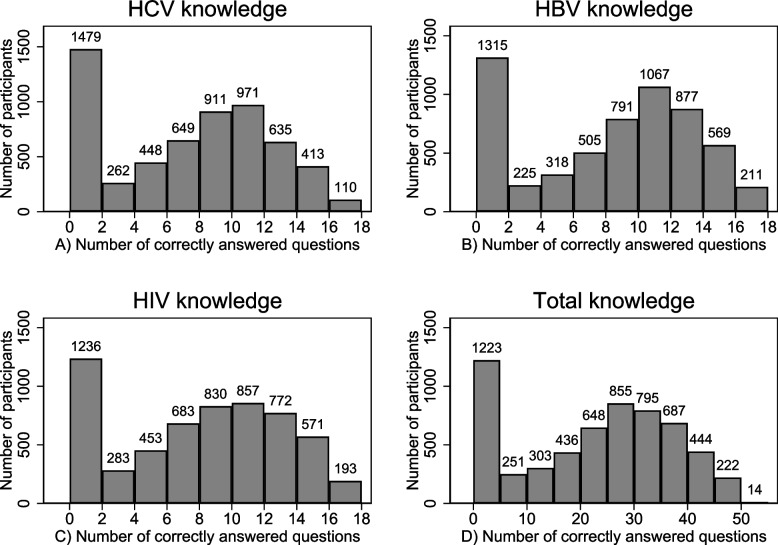
Overall knowledge score for HBV, HCV and HIV and total overall knowledge score. Overall knowledge score evaluates all 17 statements of each infection and total overall knowledge score evaluates the sum of the overall knowledge scores of HBV, HCV and HIV (51 statements)

Cronbach’s alpha coefficient for the 17-item scale was 0.951 for HCV, 0.953 for HBV and 0.961 for HIV. The distribution of the overall knowledge score for HBV, HCV and HIV is shown in Fig. 1. For all three infections, the distribution had a mass point at 0 correctly answered questions, mainly reflecting the large number of participants replying “don’t know” or “don’t’ answer”. The median (IQR) overall score was 9 (4,12), 8 (3,11) and 9 (4,12) for HBV, HCV and HIV respectively; the corresponding figures for the transmission mode knowledge score were 4 (2, 5), 4 (2, 5), and 4 (3, 5) respectively, whereas for the misconceptions score 1 (0, 2) for all three infections.

**Fig. 2:**
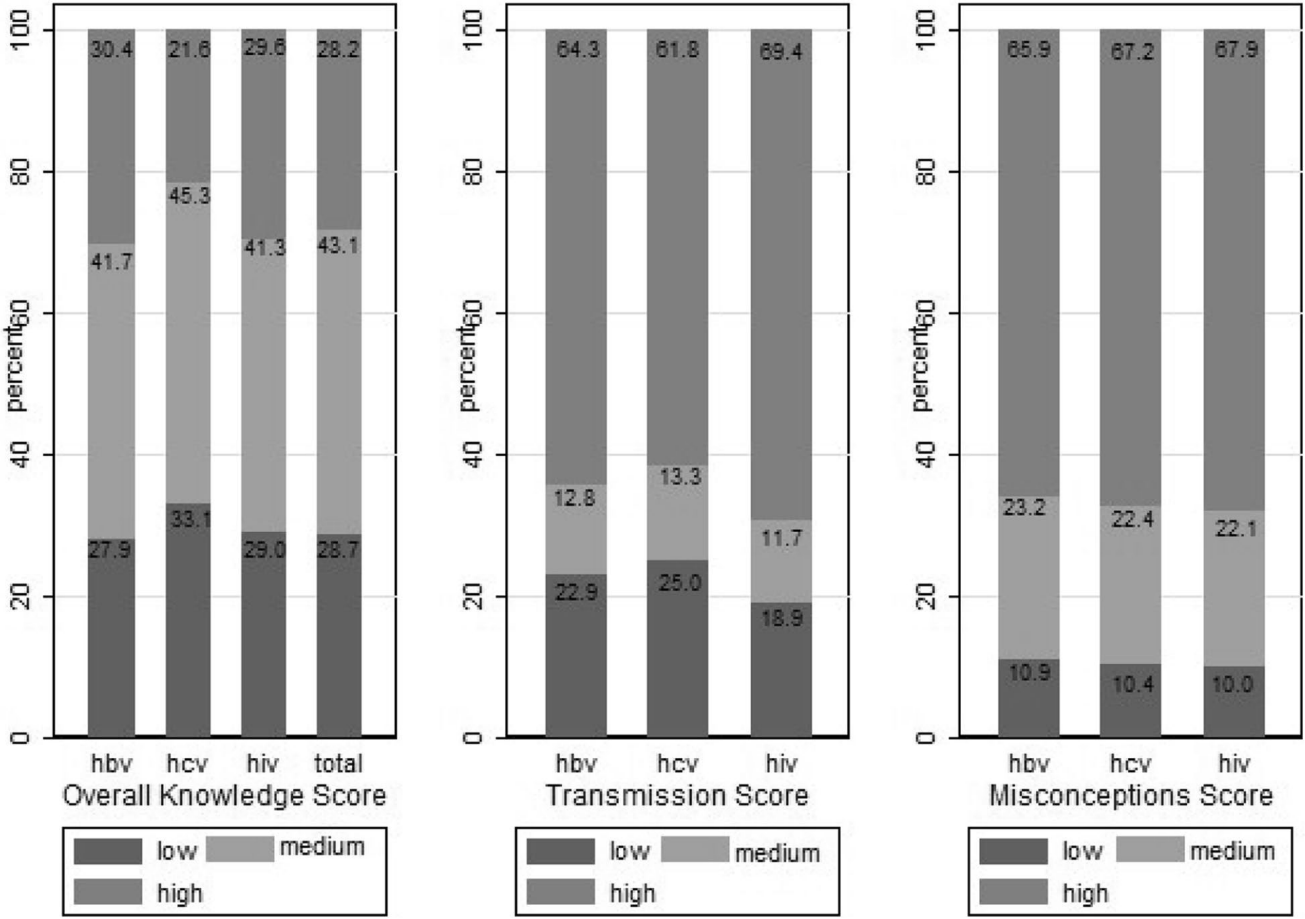
Overall, Transmission Modes and Misconceptions’ Knowledge Score for HBV, HCV, and HIV. Transmission modes knowledge score evaluates the 5 statements referring to the true routes of transmission and misconceptions’ score evaluates the 5 statements referring to false transmission routes

The overall knowledge for HBV was low for the 27.9% (1,858) of the study population, moderate for 41.7% (2,360) and high for the 30.4% (1,660). The corresponding percentages for HCV were 33.1% (2,188), 45.3% (2,517), 21.6% (1,173) and for HIV 29.0% (1,971), 41.3% (2,348), 29.6% (1,559). The level of knowledge on the modes through which these three infectious diseases are actually transmitted was generally high: 64.3% for hepatitis B, 61.8% for hepatitis C, and 69.4% for HIV. However, the misconceptions score was also high reaching 65.9% for HBV, 67.2% for HCV, and 67.9% for HIV (Fig. 2). The median (IQR) total overall knowledge score (i.e., across all 3 infections) was 26 (13, 35). In total, 1,927 (28.7%) were classified as having low total overall score, 2,448 (43.1%) as having moderate and 1,503 (28.2%) as having high score.

### Medical and behavior scores

Responses to individual items for the medical and behavior score are presented in Annex 2. The percentage of missing values (including unknown/no answer) in individual items ranged from 0.9% to 15.4% for medical and from 8.1% to 28.7% for the behavior score. Imputing missing data led to broadly similar percentages to those obtained after restricting analysis to those with valid responses (Annex 2). The most common behaviors associated with increased risk of infection were “not usually using condom” and “tattoo or piercing” as single factors or in combination, often also combined with “diagnosis of other sexually transmitted diseases”. For medical score, the most common risk factor was “surgery with narcosis or endoscopy” followed by “family medical history” and “occupational risk”; combination of two of them was the most common pattern of two co-existing factors. In Table 2, total medical and total behavior scores categorized in 3 levels are shown. Under “unknown” are included all participants with missing values in at least one item. Restricting the analysis to those with replies to all items, a minority presented with high medical (8.6%) or behavior (14.7%) score. The percentages were similar after imputing missing values in each individual item.

**Table 2:** Risk Factors Score. Total medical and behavior risk scores are categorized in three levels. All participants with missing values in at least one item are included in “Unknown”

	**N (% overall)**^a^	**%**^a^** (excluding those with missing)**	**%**^a^** after MI**^b^
**Total medical risk score**			
Low	1,307 (25.1)	34.0	33.8
Moderate	2,654 (42.4)	57.4	57.6
High	409 (6.3)	8.6	8.6
Unknown	1,508 (26.1)	-	-
**Total behavior risk score**			
Low	747 (16.4)	30.5	26.6
Moderate	1,771 (29.5)	54.8	61.1
High	436 (7.9)	14.7	12.3
Unknown	2924 (46.1)	-	

^a^weighted percentages

^b^*MI* Multiple imputations

### Factors associated with having high total overall knowledge score

In Table 3 the results from the final multivariable ordinal logistic model for total overall knowledge score after imputing missing data of the investigated co-factors are presented. Among all factors investigated, age, gender, educational level, residence area, family monthly income, country of birth, past examination for at least one infection and medical and behavior scores were significantly associated with the total overall knowledge score. Briefly, women, those born in Greece/Cyprus, and those who had been examined in the past for at least one infection were more likely to have higher knowledge levels. Those living out of the big metropolitan cities (Athens/Thessaloniki) were less likely to have higher knowledge levels whereas the older the participants, the less likely to have higher knowledge levels. There was a tendency for as higher knowledge level as higher the educational level and as larger the family monthly income. Medical score was strongly associated with knowledge level, with those having higher medical score being more likely to have higher knowledge level. On the contrary, there was not such a trend for behavior score; only those with moderate behavior score seem to have lower knowledge level compared to those with low behavior score. Sensitivity analysis restricting to complete cases, gave similar results (data not shown).

**Table 3:** Results from the final multivariable ordinal logistic regression model to assess factors associated with total overall knowledge score (low, moderate, high) after imputing missing data by multiple imputations

	**OR (95% CI)**	***P*-value**
**Age (per 5 years)**	.881 (.863—.904)	** < 0.0001**
**Gender** (Male^a^)	1	
Female	1.15 (1.03—1.28)	**0.013**
**Educational level** (Up to primary^a^)	1	
Up to secondary/post-secondary	2.85 (2.39—3.39)	** < 0.0001**
University or higher	5.05 (4.04—6.32)	** < 0.0001**
**Residence area** (Athens/Thessaloniki^a^)	1	
Rest of Greece	0.80 (0.68—.94)	**0.008**
**Family Monthly Income** (Up to 900€^a^)	1	
900€—1700€	1.39 (1.19—1.63)	** < 0.0001**
> 1700€	1.50 (1.20—1.88)	**0.001**
**Country of birth** (Other^a^)	1	
Greece / Cyprus	2.19 (1.78 – 2.69)	** < 0.0001**
**Past examination for at least 1 infection** (I've never been tested^a^)	1	
I was tested for at least 1 infection	1.82(1.58—2.10)	** < 0.0001**
**Behaviour Risk score** (Low^a^)	1	
Moderate	0.84 (0.72 – 0.98)	**0.029**
High	1.03 (0.81 – 1.31)	0.793
**Medical Risk score** (Low^a^)	1	
Moderate	1.24 (1.09—1.42)	**0.001**
High	1.70 (1.33—2.17)	** < 0.0001**

^a^Reference category

## Discussion

Hprolipsis health survey enabled us to document, for the first time in Greece, the general population level of knowledge on HBV, HCV and HIV infections. The results showed that there are significant gaps in the knowledge about these infectious among adults living in Greece. Although the level of knowledge regarding the modes through which these infections are transmitted were found to be high in a substantial percentage of the participants (64.3% for HBV, 61.8% for HCV, and 69.4% for HIV) even higher were the percentages of participants with high levels of misconceptions (65.9% for HBV, 67.2% for HCV, and 67.9% for HIV) with participants believing that these infections can be transmitted through daily social contact, dishwashing, toilet sharing, kissing and mosquito bites. Misconceptions lead to persisting social stigma and discrimination [12, 14–16]. Correct information and understanding helps improving the likelihood of behavioral change [33]. Lack of adequate knowledge has been recognized as a major barrier to prevention [10]. Raising thus awareness will help identifying infected individuals in need of treatment, ultimately leading to control the epidemics.

We found that women had increased odds of having higher knowledge level compared to men, a finding reported also by a study on the French population [31]. The higher knowledge level in women may be associated with prenatal testing. Older age was associated with lower odds of having high knowledge level. Several other studies have also found that younger people have a better understanding of how these infectious diseases are transmitted, most likely due to their greater and easier access to information [12, 16, 34, 35].

In our study, we found that the socio-economic status was significantly associated with knowledge level; higher educational level and greater family income were independently associated with higher knowledge level. These associations have been reported in several other studies [16, 19, 31, 34, 36–39]. Additionally, in Hprolipsis, residence in the two major urban centers of the country (Athens/Thessaloniki) and origin from Greece/Cyprus (as compared to migrants mainly from Albania or from Eastern Europe) were significantly associated with higher knowledge level. These factors are also related to the socio-economic status and to easier and more direct access to information.

The significant association, found in our study, between moderate level of behavior score and lower knowledge level may indicate the lack of awareness of these behavioral factors as being associated with higher risk, whereas the association of higher medical score with higher knowledge level may be, at least partly, explained by the more often contact of people with high medical score with health services, thus, having higher access to health information.

The substantial gap in hepatitis and HIV infections knowledge points to the urgent need for developing and implementing population educational programs. This is even more urgent in the era of COVID-19 epidemic, as it has been shown that COVID-19 epidemic may have disrupted hepatitis and HIV prevention and treatment services [40–42] whereas it may have widened health and economic inequalities [43]. However, educational programs must be targeted to be cost-effective. Based on our results, the least privileged population, namely those of low educational level and socioeconomic status, living out of big cities and migrants, should be targeted. Educational programs have to be localized and taking into account target population characteristics, habits and needs.

## Limitations

First, as in most health surveys, certain vulnerable populations such as people who inject drugs (PWID), homeless, migrants and Roma living in Roma settlements, may have been underrepresented in the general population part of the Hprolipsis study. In addition, institutionalized persons were excluded from Hprolipsis by its design. Including also these people would affect knowledge level estimates, though to an uncertain direction as some (like PWID) may have higher and others (e.g. homeless, Roma) lower knowledge level. Second, as in all cross-sectional health surveys, missing data in key variables existed. Regarding knowledge score, only 115 individuals (1.9%) were excluded from the analysis as having missing values at ≥ 6 out of the 51 knowledge questions and this exclusion could hardly have any impact on our results. Among those included in the analysis (5,878), the proportion of missing values in individual items was relatively small (around 7% in total) and our approach of imputing missing values based on the average score over valid responses for each infection should not have an impact on the results. Higher percentages of missing values were observed in behavior and, to a lesser extent, medical risk score items. However, we implemented the MI approach to fill in missing item responses and results after imputing missing values were similar to those of the complete case analysis. Third, we evaluated knowledge level based on a quantitative questionnaire. Semi-structured interviews with open questions may had provided a more in depth insight on participants’ knowledge and awareness.

## Conclusion

Hprolipsis study enabled us to document, for the first time in Greece, the knowledge of the general population on HBV, HCV and HIV infections. The results showed that there are significant gaps in adults living in Greece knowledge level, with high rates of misbeliefs. Our study identified several socio-economic (low educational level, low family income, migrants from Albania/Eastern Europe), and demographic (male gender, residence out of major urban centers) risk factors associated with lower knowledge on HIV, HBV and HCV infections. Consequently, tailored interventions can be implemented to enhance knowledge level on infectious diseases among specific subgroups of the general population. Innovative approaches, in collaboration with municipalities (particularly those being far from metropolitan cities), migrants’ and Roma communities, organizations against drugs and other relevant non-governmental organizations, should be designed and implemented targeting the least privileged population, in principle at higher risk for these infections. The general population could be reached/informed by primary care physicians, provided such program is in place. Accurate knowledge could also contribute in decreasing stigmatization and discrimination, increasing detection rates and identification of patients in need of therapy, ultimate facilitating control of the infections’ epidemics. Public HIV-awareness campaigns led to reduced stigma and discrimination toward individuals living with HIV infection [44]. There is thus an urgent need for large scale but also localized targeted to less privileged populations awareness activities to fill the gaps in knowledge, increase population engagement in preventive measures, reduce the likelihood of discrimination and prejudice and improve adherence to treatment.

## Supplementary Information

**Additional file 1:** **Annex 1. **Knowledge of HBV, HCV and HIV in the general adult Greek population: Questions and replies. **Annex 2. **Risk Factors Questions and replies.

## Data Availability

The datasets generated and/or analysed during the current study are not publicly available as they contain sensitive personal health data but are available from the corresponding author on reasonable request.
