# Supplementary material for: Hepatitis B, C and human immunodeficiency virus knowledge among the general greek population: results from the Hprolipsis nationwide survey
Source: BMC Public Health. 2022 Nov 5;22:2026. doi: 10.1186/s12889-022-14353-9 (PMC9637311; doi:10.1186/s12889-022-14353-9)
Supplement: Supplementary file 1 — Additional file 1: Annex 1. Knowledge of HBV, HCV and HIV in the general adult Greek population: Questions and replies. Annex 2. Risk Factors Questions and replies. [file 12889_2022_14353_MOESM1_ESM.docx]

**ANNEX 1: Knowledge of HBV, HCV and HIV (Ν=5878): Questions and replies [N (% weighted)]. Correct answers are indicated in bold.**

General question: *We would like to tell us which of the arguments presented below for hepatitis B, C and HIV/AIDS are right or wrong. Please do not try to guess the correct answer if you don’t know it. You can reply “I don’t know” or “I don’t answer”*. Correct answers are indicated in bold**.**

| **Question** | **Right**  **[N (%)]** | **Wrong**  **[N (%)]** | **Unknown**  **[N (%)]** | **Don’t know**  **[N (%)]** | **Don’t answer [N (%)]** |
| --- | --- | --- | --- | --- | --- |
| **Hepatitis B** | | | | | |
| 1.Someone with hepatitis B can transmit the virus to someone else | **3,558 (60.53)** | 169 (2.88) | - | 2,011 (34.21) | 140 (2.38) |
| 2.A person may have hepatitis B virus for all his life | **2,149 (36.56)** | 531 (9.03) | 10 (0.17) | 3,042 (51.75) | 146 (2.48) |
| 3.If someone has hepatitis B but looks and feels healthy, he/she cannot transmit the virus to another person | 993 (16.89) | **1,829 (31.12)** | 4 (0.07) | 2,907 (49.46) | 145 (2.47) |
| 4.Hepatitis B can lead to cirrhosis or liver cancer | **2,471 (42.04)** | 254 (4.32) | 7 (0.12) | 2,998 (51.00) | 148 (2.52) |
| 5.The only way for someone to find out if he/she has hepatitis B, is to perform the corresponding test | **3,166 (53.86)** | 271 (4.61) | 3 (0.05) | 2,292 (38.99) | 146 (2.48) |
| 6.Hepatitis B infection is preventable by vaccination | **2,854 (48.55)** | 190 (3.23) | 7 (0.12) | 2,687 (45.71) | 140 (2.38) |
| 7. There is treatment for hepatitis B | **2,154 (36.65)** | 449 (7.64) | 7 (0.12) | 3,123 (53.13) | 145 (2.47) |
| **Hepatitis B is transmitted by:** | | | | | |
| 8.Infected blood transfusion | **4,302 (73.19)** | 38 (0.65) | 1 (0.02) | 1,343 (22.85) | 194 (3.30) |
| 9.Sexual contact without a condom | **4,079 (69.39)** | 100 (1.70) | 3 (0.05) | 1,501 (25.54) | 195 (3.32) |
| 10.From mother to embryo | **2,792 (47.50)** | 343 (5.84) | 4 (0.07) | 2,526 (42.97) | 213 (3.62) |
| 11.Tattoos or body piercing | **3,418 (58.15)** | 278 (4.73) | 3 (0.05) | 1,964 (33.41) | 215 (3.66) |
| 12.Injection drug use | **4,091 (69.60)** | 76 (1.29) | 1 (0.02) | 1,500 (25.52) | 210 (3.57) |
| **Misconceptions about transmission modes of hepatitis B (Hepatitis B is transmitted by:)** | | | | | |
| 13.Every day encounters (hand shaking, conversation) | 383 (6.52) | **3,242 (55.15)** | 15 (0.26) | 2,022 (34.40) | 216 (3.67) |
| 14.Drinking or eating from the same utensils as someone infected | 1,359 (23.12) | **1,810 (30.79)** | 13 (0.22) | 2,477 (42.14) | 219 (3.73) |
| 15.Using the same toilet, pool, sauna with someone with infection | 1,788 (30.42) | **1,401 (23.83)** | 7 (0.12) | 2,465 (41.94) | 217 (3.69) |
| 16.Kiss | 1,756 (29.87) | **1,403 (23.87)** | 9 (0.15) | 2,490 (42.36) | 220 (3.74) |
| 17.Mosquito bite | 1,664 (28.31) | **1,228 (20.89)** | 14 (0.24) | 2,755 (46.87) | 217 (3.69) |
| **Hepatitis C** | | | | | |
| 1.Someone with hepatitis C can transmit the virus to someone else | **3,076 (52.33)** | 161 (2.74) | 2 (0.03) | 2,496 (42.46) | 143 (2.43) |
| 2.A person may have hepatitis C virus for all his life | **1,814 (30.86)** | 483 (8.22) | 14 (0.24) | 3,422 (58.22) | 145 (2.47) |
| 3.If someone has hepatitis C but looks and feels healthy, he/she cannot transmit the virus to another person | 911 (15.50) | **1,634 (27.80)** | 8 (0.14) | 3,180 (54.10) | 145 (2.47) |
| 4.Hepatitis C can lead to cirrhosis or liver cancer | **2,208 (37.56)** | 190 (3.23) | 10 (0.17) | 3,322 (56.52) | 148 (2.52) |
| 5.The only way for someone to find out if he/she has hepatitis C, is to perform the corresponding test | **2,888 (49.13)** | 274 (4.66) | 5 (0.09) | 2,565 (43.64) | 146 (2.48) |
| 6.Hepatitis C infection is preventable by vaccination | 1,807 (30.74) | **518 (8.81)** | 10 (0.17) | 3,401 (57.86) | 142 (2.42) |
| 7.There is treatment for hepatitis C | **1,738 (29.57)** | 549 (9.34) | 15 (0.26) | 3,430 (58.35) | 146 (2.48) |
| **Hepatitis C is transmitted by:** | | | | | |
| 8.Infected blood transfusion | **4,112 (69.96)** | 37 (0.63) | 6 (0.10) | 1,528 (26.00) | 195 (3.32) |
| 9.Sexual contact without a condom | **3,916 (66.62)** | 98 (1.67) | 10 (0.17) | 1,658 (28.21) | 196 (3.33) |
| 10.From mother to embryo | **2,689 (45.75)** | 349 (5.94) | 12 (0.20) | 2,614 (44.47) | 214 (3.64) |
| 11.Tattoos or body piercing | **3,286 (55.90)** | 280 (4.76) | 4 (0.07) | 2,092 (35.59) | 216 (3.67) |
| 12.Injection drug use | **3,978 (67.68 )** | 78 (1.33) | 3 (0.05) | 1,609 (27.37) | 210 (3.57) |
| **Misconceptions about transmission modes of hepatitis C (Hepatitis C is transmitted by:)** | | | | | |
| 13.Every day encounters (hand shaking, conversation) | 366 (6.23) | **3,150 (53.59)** | 18 (0.31) | 2,127 (36.19) | 217 (3.69) |
| 14.Drinking or eating from the same utensils as someone infected | 1,278 (21.74) | **1,777 (30.23)** | 14 (0.24) | 2,589 (44.05) | 220 (3.74) |
| 15.Using the same toilet, pool, sauna with someone with infection | 1,723 (29.31) | **1,362 (23.17)** | 9 (0.15) | 2,564 (43.62) | 220 (3.74) |
| 16.Kiss | 1,686 (28.68) | **1,372 (23.34)** | 15 (0.26) | 2,585 (43.98) | 220 (3.74) |
| 17.Mosquito bite | 1,586 (26.98) | **1,216 (20.69)** | 15 (0.26) | 2,843 (48.37) | 218 (3.71) |
| **HIV/ AIDS** | | | | | |
| 1.When someone has HIV, has necessarily symptoms | 1,262 (21.47) | **1,658 (28.21)** | 9 (0.15) | 2,666 (45.36) | 283 (4.81) |
| 2.If someone has HIV/AIDS but looks and feels healthy, he/she cannot transmit the virus | 1,080 (18.37) | **2,181 (37.10)** | 6 (0.10) | 2,332 (39.67) | 279 (4.75) |
| 3.HIV infections can cause a decline in the body’s defense | **3,241 (55.14)** | 145 (2.47) | 9 (0.15) | 2,206 (37.53) | 277 (4.71) |
| 4.The only way for someone to learn if he/she is positive, is to do the test | **2,258 (38.41)** | 847 (14.41) | 13 (0.22) | 2,484 (42.26) | 276 (4.70) |
| 5.There is vaccination for HIV/AIDS | 898 (15.28) | **1,664 (28.31)** | 40 (0.68) | 2,996 (50.97) | 280 (4.76) |
| 6.There is treatment for HIV/AIDS | **2,307 (39.25)** | 927 (15.77) | 20 (0.34) | 2,347 (39.93) | 277 (4.71) |
| 7.(If the previous answer is “True”) The treatments can definitively cure from the HIV infection | 743 (32.21) | **1,088 (47.16)** | 9 (0.39) | 465 (20.16) | 2 (0.09) |
| **HIV is transmitted by:** | | | | | |
| 8.Infected blood transfusion | **4,467 (76.00)** | 38 (0.65) | 10 (0.17) | 1,169 (19.89) | 194 (3.30) |
| 9.Sexual contact without a condom | **4,507 (76.68)** | 42 (0.71) | 13 (0.22) | 1,122 (19.09) | 194 (3.30) |
| 10.From mother to embryo | **3,052 (51.92)** | 315 (5.36) | 18 (0.31) | 2,280 (38.79) | 213 (3.62) |
| 11.Tattoos or body piercing | **3,509 (59.70)** | 313 ( 5.32) | 16 (0.27) | 1,826 (31.06) | 214 (3.64) |
| 12.Injection drug use | **4,337 (73.78)** | 67 (1.14) | 5 (0.09) | 1,266 (21.54) | 203 (3.45) |
| **Misconceptions about transmission modes of HIV (HIV is transmitted by:)** | | | | | |
| 13.Every day encounters (hand shaking, conversation) | 358 (6.09) | **3,430 (58.35)** | 22 (0.37) | 1,853 (31.52) | 215 (3.66) |
| 14.Drinking or eating from the same utensils as someone infected | 1,255 (21.35) | **2,079 (35.37)** | 35 (0.60) | 2,291 (38.98) | 218 (3.71) |
| 15.Using the same toilet, pool, sauna with someone with infection | 1,536 (26.13) | **1,739 (29.58)** | 25 (0.43) | 2,363 (40.20) | 215 (3.66) |
| 16.Kiss | 1,786 (30.38) | **1,582 (26.91)** | 24 (0.41) | 2,269 (38.60) | 217 (3.69) |
| 17.Mosquito bite | 1,661 (28.26) | **1,335 (22.71)** | 29 (0.49 ) | 2,637 (44.86) | 216 (3.67) |

**Annex 2: Risk Factors Questions and replies [N (% weighted)].** *Weighted percentages are provided prior to and after filling in missing responses through multiple imputations.*

|  | N (%) Overall | | | % (excluding those with missing) | | % after MI^&^ | |
| --- | --- | --- | --- | --- | --- | --- | --- |
|  | **No** | **Yes** | **NΑ/**  **Unknown** | **No** | **Yes** | **No** | **Yes** |
| **Medical Risk** |  |  |  |  |  |  |  |
| During your work, do you come in contact with blood, blood products, syringes or needles? | 4,719 (79.68) | 281 (4.88) | 878 (15.43) | 94.23 | 5.77 | 94.33 | 5.67 |
| Have you ever undergone surgery requiring anesthetization? | 2,176 (41.14) | 3,644 (57.96) | 58 (0.90) | 41.51 | 58.49 | 41.59 | 58.42 |
| Have you ever undergone endoscopy? | 4,163 (73.93) | 1,579 (23.91) | 136 (2.16) | 75.56 | 24.44 | 75.62 | 24.83 |
| Have you ever undergone dialysis? | 5,734 (97.7) | 9 (0.15) | 135 (2.15) | 99.85 | 0.16 | 99.81 | 0.19 |
| Has any member of your household been diagnosed with hepatitis B or C? | 5,082 (86.69) | 131 (2.02) | 665 (11.29) | 97.72 | 2.28 | 97.69 | 2.31 |
| Have you had a blood transfusion (before 1992)? | 5,534 (94.79) | 226 (3.35) | 118 (1.86) | 96.59 | 3.41 | 96.58 | 3.42 |
| Have you ever had an organ transplant? | 5,709 (97.26) | 19 (0.32) | 150 (2.42) | 99.67 | 0.33 | 99.62 | 0.38 |
| **Behavioural Risk** |  |  |  |  |  |  |  |
| Have you had tattoos or body piercing recently or in the past? | 4,008 (67.22) | 677 (12.92) | 1,193 (19.86) | 83.87 | 16.13 | 84.15 | 15.85 |
| During the last year have you had more than 5 new sexual partners? | 4,604 (78.0) | 66 (1.75) | 1,208 (20.24) | 97.8 | 2.20 | 97.20 | 2.80 |
| Have you ever been diagnosed with a sexually transmitted disease? | 5,133 (86.92) | 256 (5.02) | 489 (8.07) | 94.54 | 5.46 | 94.72 | 5.28 |
| Do you usually use condom? | 2,583 (40.79) | 1,385 (30.57) | 1,910 (28.65) | 57.16 | 42.84 | 62.36 | 37.64 |
| Have you injected drugs recently or in the past? | 4,723 (80.36) | 27 (0.66) | 1,128 (18.98) | 99.19 | 0.81 | 98.92 | 1.08 |
| *NA: No Answer;* ^&^*MI: Multiple imputations* | | | | | | | |
